# Evaluating the accuracy of the MBT Lipid Xtract Kit for assessing colistin resistance in comparison to broth microdilution

**DOI:** 10.1099/jmm.0.001881

**Published:** 2024-09-02

**Authors:** Calvin Ka-Fung Lo, Gordon Ritchie, Jennifer Bilawka, Leah Gowland, Samuel D. Chorlton, Willson Jang, Nancy Matic, Marc G. Romney, Aleksandra Stefanovic, Christopher F. Lowe

**Affiliations:** 1 Department of Pathology and Laboratory Medicine, University of British Columbia, Vancouver, British Columbia, Canada; 2 Division of Medical Microbiology and Virology, Providence Health Care, St. Paul’s Hospital, Vancouver, British Columbia, Canada; 3 BugSeq Bioinformatics, Vancouver, British Columbia, Canada

**Keywords:** BMD, colistin, lipidomics, *mcr-1*, resistance screening, susceptibility testing

## Abstract

Colistin resistance testing methods such as broth microdilution (BMD) are time-consuming and labour intensive for clinical laboratories. MBT Lipid Xtract Kit on MALDI Biotyper Sirius System (Bruker, Billerica, MA, USA) utilizes lipidomic analysis to identify specific cell wall modifications associated with colistin resistance. We compared MBT to BMD (ComASP Colistin, Liofilchem) across 36 Gram-negative isolates (non-resistant MIC ≤2 µg ml^−1^, resistant MIC ≥4 µg ml^−1^). All samples were tested twice on MBT with discrepant results repeated before assessing categorical agreement between MBT and BMD. 44.4% (16/36) of isolates were colistin resistant via BMD. MBT Lipid Xtract had 80.6% agreement (29/36) with BMD, with 5/7 discrepancies corrected to match upon repeat testing. There was 100% agreement for *Escherichia coli* isolates (*n*=16). The whole-genome sequencing was completed on the two discrepant *Klebsiella pneumoniae* isolates, with variants within colistin resistance-associated loci identified (MIC 0.5 µg ml^−1^: *arnC* S30T, *pmrB* T246A, *lapB* N212T, *lpxM* S253G, *crrB* Q287K and MIC >16 µg ml^−1^: *arnC* S30T, *pmrB* R90insRN, *pmrB* T246A, *pmrA* E57G, *lpxM* S253G). Further evaluation, particularly for non-*E. coli*, of MBT is required prior to implementation in clinical laboratories.

**Impact Statement**

Amidst the current limited access to antimicrobials such as ceftazidime-avibactam and cefiderocol, colistin has re-emerged as an alternative therapeutic option for multi-drug-resistant Gram-negative infections. However, current testing for colistin resistance remains challenging due to long turnaround times and technical expertise beyond routine laboratory practice. MALDI Biotyper Sirius System (Bruker, Billerica, MA, USA) can detect changes in bacterial lipid structures that are predictive for antibiotic resistance, including colistin. Furthermore, its software module (LipidART) detects specific charge patterns to determine whether resistance is based on chromosomal or plasmid-coded modifications, the latter of which being more associated with patient-to-patient transmission. Overall, with MBT Lipid Xtract Kit, analytical testing only requires 30 min from extraction of bacterial colony to report generation. As the first Canadian academic site evaluating MBT Lipid Xtract Kit, we conducted a pilot study comparing colistin screening results against broth microdilution.

## Data Availability

Sequence data has been uploaded to GenBank (BioProject number PRJNA1150639).

## Introduction

With the global proliferation of multi-drug-resistant Gram-negative bacteria, such as carbapenemase-producing *Enterobacterales* (CPE), effective treatment options are often limited [1]. In Canada, CPE have progressively increased from 0.03 to 0.05 infections per 10 000 patient days between 2016 and 2020, with concern for transmission within healthcare facilities [2]. Novel antimicrobials have been developed in response to combat carbapenem-resistant pathogens such as ceftazidime-avibactam, though some novel agents do not cover all CPE, specifically those harbouring metallo-β-lactamases [3]. Given the limited access to these novel antimicrobials and varying mechanisms of carbapenem resistance, colistin has re-emerged as a potential complementary antimicrobial of last resort for combatting multi-drug-resistant Gram-negative infections.

Colistin resistance may be intrinsically mediated but can also stem from acquired mechanisms, mostly from modifications of the LPS drug target. These modifications correspond to the addition of cationic groups such as 4-amino-l-arabinose and/or phosphoethanolamine on lipid A, an LPS anchor. These LPS modifications have also been observed in plasmid-mediated transmission of the mobilized colistin resistance (*mcr*) genes across different *Enterobacterales* species [4]. Concern for broad dissemination of colistin resistance through plasmid-mediated transfer has been reported for *mcr-1*, though it remains uncommon in Canada [5,6]. Rapid and accurate detection of colistin resistance is crucial to provide alternative active antibiotics for patients with severe infections due to multi-drug-resistant Gram-negative bacteria.

The recommended protocol for colistin resistance testing includes broth microdilution (BMD), broth disc elution and agar dilution. However, limitations exist with these methods, including long turnaround times greater than 24 h, extensive technical labour and specialized expertise beyond routine laboratory practice [7,8]. Agar diffusion remains challenging given the amphophilic nature of colistin, resulting in difficulties in resistance interpretation of small inhibition zones [9]. Gradient diffusion strips have very high major error rates when compared to BMD and are not a recommended testing modality [7,10]. Automated susceptibility platforms (e.g. Vitek2) also have similar high error rates [11].

Lipidomics have been utilized to identify cell wall modifications predictive of antibiotic resistance, such as the negative ion mode on the MALDI Biotyper Sirius System (MBT) [12]. Matrix-assisted laser desorption/ionization-time of flight (MALDI-ToF) spectra are acquired via the dedicated LipidART software module, which provides interpretations on whether resistance is based on chromosomal or plasmid-coded modifications via detection of specific mass to charge ratio (*m/z*) peaks [13]. In contrast to BMD, colistin resistance testing with MBT only requires 30 min after bacterial colonies have been isolated.

We conducted a pilot study comparing colistin screening results of MBT Lipid Xtract Kit against the reference standard (BMD) across various Gram-negative bacterial isolates.

## Methods

Gram-negative isolates identified between 2020 and 2022, which were previously tested for colistin resistance by a commercial BMD assay (ComASP Colistin, Liofilchem) at St. Paul’s Hospital Microbiology Laboratory (Vancouver, BC), were included. The interpretation of colistin non-susceptibility was based upon Clinical & Laboratory Standards Institute (CLSI) breakpoints for *Enterobacterales*, *Pseudomonas aeruginosa* and *Acinetobacter* spp.; colistin non-resistance included organisms with MIC ≤2 µg ml^−1^ and resistance with MIC ≥4 µg ml^−1^ [7]. In addition, we also included information regarding any other resistance mechanisms noted from historical laboratory data records [e.g. presence of AmpC β-lactamase, extended-spectrum β-lactamase (ESBL) and carbapenemase]. Organisms intrinsically resistant to colistin (e.g. *Morganella* spp., *Proteus* spp., *Providencia* spp. and *Serratia* spp.) were excluded.

Colonies derived from frozen isolates were sub-cultured three times, and then, colonies from the third sub-culture incubated for 18 h were suspended with 50 µl of hydrolysis buffer in 1.5-ml microtube prior to discarding 44 µl. The remaining volume was heated at 90 °C for 10 min before allowing to dry for 2 min. The tube was subsequently placed at room temperature with 50 µl of washing buffer added. Vortexing was completed for 1 s to remove interfering molecules before removal of the washing buffer. Five microlitres of the matrix was then added to the remaining material for resuspension before depositing 2 µl of the sample directly on the MBT target plate. Each isolate was tested twice on MBT under negative ion mode as per the manufacturer’s recommendation by one senior laboratory technologist. As per the manufacturer’s recommendations, if there were discordant results among the duplicate testing, a repeat MBT test was performed. Isolates with MBT results discrepant from BMD were also repeated on a subsequent run to confirm the initial MBT result.

Isolates with discordant results between BMD and MBT underwent whole-genome sequencing (WGS). The extraction was performed on the MagNA Pure 24 (Roche Diagnostics). Sequencing was conducted on the GridION (Oxford Nanopore Technologies) with R10.4.1 flowcells, and Raw FASTQ files were analysed by BugSeq [14]. Sequences were analysed for the following genes or amino acid substitutions therein associated with colistin resistance and identified in recent reviews: *mcr*, *arnC*, *crrB*, *lapB*, *lpxM*, *mgrB*, *phoP*, *phoQ*, *pmrA* and *pmrB* [4].

The categorical agreement was computed between MBT and BMD MIC interpretation. A separate analysis was also completed for non-*Escherichia coli* isolates, including manual analysis of MALDI-ToF spectra values.

## Results

A total of 36 isolates were gathered from our available antibiotic resistance database. Per BMD, 16/36 (44.4 %) had MIC values >2 µg ml^−1^, i.e. colistin resistant. Twenty of those isolates had other noted mechanisms of antibiotic resistance including CPE, ESBL or AmpC. Isolates were predominantly *E. coli* and *Klebsiella pneumoniae* (Table 1). Three of the 16 *E. coli* and 9/12 *K*. *pneumoniae* had recorded colistin MICs ≥4 µg ml^−1^ via BMD.

**Table 1.** Characteristics of included isolates (*n*=36)

Organism	Colistin resistance by BMD	AmpC β-lactamase	ESBL	Carbapenemase
*E. coli* (16, 44.4%)	3*	3	3	6
*Enterobacter cloacae* complex (3, 8.3%)	2	3	0	2
*K. pneumoniae* (12, 33.3%)	9	1	6	2
*Citrobacter freundii* complex (2, 5.6%)	0	2	0	2
*Acinetobacter baumannii* complex (1, 2.8%)	1	0	0	0
*P. aeruginosa* (1, 2.8%)	0	0	0	0
*K. aerogenes* (1, 2.8%)	1	1	0	0

*1One isolate was confirmed as *mcr-1*.

Twenty-nine of the 36 isolates (80.6%) tested on MBT matched BMD results; there was 100% agreement with all *E. coli* isolates. There were seven discordant isolates including six *K*. *pneumoniae* and one *Klebsiella aerogenes* (Table 2). Repeat testing on MBT was conducted as per manufacturer recommendations due to discordance in the first run (NR/error, NR/R or R/NR). In three of the four cases, repeat MBT testing produced concordant results (NR/NR or R/R) which correlated with BMD, while one case (sample 1), which had an MIC >16 µg ml^−1^, continued to produce uninterpretable results (NR/error, error/R). To further investigate, WGS was performed on this sample and identified potential amino acid substitutions associated with colistin resistance (*arnC* S30T, *pmr*B R90RN *pmrB* T246A, *pmrA* E57G and *lpxM* S253G).

**Table 2.** Analysis of discordant results via MBT Lipid Xtract Kit (*n*=7)

Sample	Organism with discordant result	BMD MIC	MBT first run	MBT repeat run	Discrepancy type	Corrected result	WGS
1	*K. pneumoniae*	>16	NR/error	Error/R	Inconsistent R result	No	*arnC* S30T*pmrB* R90RN*pmrB* T246A*pmrA* E57G*lpxM* S253G
2	*K. pneumoniae*	0.25	NR/R	NR/NR	Discrepant R/NR	Yes	
3	*K. pneumoniae*	8	R/NR	R/R	Discrepant R/NR	Yes	
4	*K. pneumoniae*	>8	R/NR	R/R	Discrepant R/NR	Yes	
5	*K. pneumoniae*	0.5	R/R	R/R	Falsely resistant	No	*arnC* S30T*pmrB* T246A*lapB* N212T*lpxM* S253G*crrB* Q287K
6	*K. pneumoniae*	4	NR/NR	R/R	Falsely non-resistant	Yes	
7	*K. aerogenes*	8	NR/NR	R/R	Falsely non-resistant	Yes	

NRnon-resistantRresistant

For the remaining three cases in Table 2, repeat MBT testing was conducted in an attempt to troubleshoot discordant MBT and BMD results. Repeat MBT for sample 5 (R/R) was unchanged and still discordant with BMD. Repeat MBT testing for samples 6 and 7 (NR/NR to R/R) corrected to the expected BMD (4 and 8 µg ml^−1^, respectively). Due to the unchanged MBT results discordant from BMD (0.5 µg ml^−1^) in sample 5, WGS was completed on this isolate, and variants within loci were associated with colistin resistance (*arnC* S30T, *pmrB* T246A, *lpxM* S253G, *lapB* N212T and *crrB* Q287K).

## Discussion

Overall, MBT demonstrated 80.6% agreement with BMD with no identified discrepancies for *E. coli* isolates. A significant majority of discrepancies (85.7%) were noted with *K. pneumoniae* isolates and, based on the manufacturer’s recommendations, were resolved on repeat testing (5/7, 71%). The reason for the discordances predominantly with *K. pneumoniae* isolates remains unclear. It was speculated that heavily mucoid colonies impaired the analysis of the lipid *m/z* values [15]; however, the isolates with discordant results were not mucoid and processed by an experienced senior technologist specifically trained on the MBT. Going forward, our laboratory will be mindful of mucoid isolates potentially affecting colistin interpretation, with specialized protocols for managing these strains (e.g. repeat MBT testing, routine duplicate MBT testing and alternative testing such as molecular testing or BMD or WGS). Another possible explanation for discordant results could be the presence of resistance mechanisms outside of lipid A modifications. For example, *K. pneumoniae* has been reported to demonstrate colistin resistance via overproduction of the surface anionic capsular polysaccharides, which serves as a protective barrier against polymyxins, concurrently with the upregulation of capsular biosynthesis genes [e.g. *siaD*, *OmpA* and *cps* operon (*wca*)], which trap polymyxins and hinder binding with lipid A [16].

Although colistin is not a recommended first-line antimicrobial, there are defined clinical situations where it may be utilized for multi-drug-resistant Gram-negative infections. Rapid colistin resistance testing can facilitate targeted use of colistin where it may potentially have activity. When the results of conventional testing (e.g. BMD) are still pending, a rapid test indicating colistin resistance may minimize colistin exposure and therefore prevent the risk of nephrotoxicity. MBT performed well for colistin resistance to *E. coli*, which was similar to a two-site study in Germany and UK reporting sensitivity and specificity at both sites exceeding 96% for *E. coli* (*n*=90) [17]. Further studies are required investigating the accuracy of MBT in non-*E. coli* isolates to enable broader application in clinical laboratories where requests for colistin resistance testing are not solely for *E. coli*.

Alternative rapid resistance screening methods have also been described including fast lipid analysis technique mass spectrometry where the lipids are extracted directly from heated surface of MALDI plate [12,18]. When compared to BMD across 98 *Enterobacter* spp. and *K. aerogenes* isolates, the sensitivity was 100% for resistant isolates (based on the detected presence of Ara4N modification). Low specificity (53.4%) was identified for isolates with MIC ≤2 µg ml^−1^ [12]. Molecular testing for colistin resistance, specifically *mcr*, has been increasingly available both as laboratory-developed tests or in commercial multiplex PCR assays [19,20]. While molecular tests can be helpful to confirm potential colistin resistance determined by MBT or BMD due to *mcr*, a negative molecular test does not rule out resistance as other mechanisms of resistance exist. Even within the plasmid-mediated *mcr* resistance mechanism, novel variants of *mcr* have been described and laboratories need to continually re-assess whether their assay is specific to *mcr-1* or covers all variants (*mcr1-10*) [21,22].

Studies utilizing WGS have highlighted the diagnostic challenges associated with colistin resistance, as well as challenges with WGS interpretation for colistin. Torres *et al.* conducted WGS analysis on Illumina Miseq 2×300 bp or NextSeq 2×150 bp for 97 isolates (54 resistant as per BMD); only four and one isolates detected *mcr-1* and *mcr-2*, respectively, with the majority of resistant isolates carrying spontaneous chromosomal amino acid substitutions (e.g. *mgrB*, *phoQ*, *pmrA*, *pmrB* and *pmrC*) [23]. However, the low sample size in this study confounded the ability to fully correlate resistance mechanisms with BMD MIC values [23]. While WGS for genomic predictors of resistance has been increasingly utilized for confirmation of antimicrobial resistance, further data are required for colistin as there are conflicting reports regarding the association between an amino acid substitution and phenotypic resistance (e.g. *pmrB* T246A and *pmrA* E57G) [24,25]. Our study identified variants in both resistant and non-resistant samples associated with resistance in the literature [4]. In addition, the interpretation can be a challenge with novel amino acid substitutions, such as *pmrB* R90RN identified in sample 1, which to our knowledge has not been described to date in literature. Resistance may require more than one amino acid substitution, or cumulative amino acid substitutions at specific loci, which may necessitate a machine learning model to accurately predict given the complexity of colistin resistance. As shown in our study, while similar amino acid substitutions were identified in samples 1 and 5, they did not correlate with phenotypic testing by BMD.

The limitations of our study included the small sample size with fewer than 40 isolates. Colistin resistance is fortunately relatively rare within Canada [5,6], and within our healthcare network, we attempted to collect as many colistin-resistant or multi-drug-resistant isolates (e.g. CPE) where colistin resistance testing may be requested. As a pilot study to assess the feasibility of the novel MBT technology in a clinical laboratory, this study was not designed as a clinical laboratory validation and a larger sample size is required to assess colistin resistance in non-*E. coli* isolates, prior to widespread application in clinical context.

The broad incorporation of MALDI-ToF into clinical laboratories has transformed the identification of micro-organisms, and novel developments in antimicrobial resistance detection with this technology may provide further utility for clinical microbiology laboratories. Our assessment of MBT Lipid Xtract Kit for colistin resistance identified concordant results for *E. coli* compared to BMD and can provide a rapid result for patients when colistin treatment is indicated. However, further research and investigation are required before clinical laboratory implementation, particularly for assessing its performance and utility in non-*E. coli* isolates.

## Abbreviations

BMD: broth microdilution
CPE: carbapenemase-producing Enterobacterales
ESBL: extended-spectrum β-lactamase
mcr: mobilized colistin resistance
WGS: whole-genome sequencing
